# 3D-printed bioceramic scaffolds for bone defect repair: bone aging and immune regulation

**DOI:** 10.3389/fbioe.2025.1557203

**Published:** 2025-03-31

**Authors:** Haoran Qi, Bo Zhang, Feng Lian

**Affiliations:** ^1^ Department of Orthopaedic Surgery, The Fourth Affiliated Hospital of Harbin Medical University, Harbin, Heilongjiang, China; ^2^ Centre for Leading Medicine and Advanced Technologies of IHM, The First Affiliated Hospital of USTC, Hefei, Anhui, China

**Keywords:** 3D printing, bioceramic scaffolds, immune microenvironment, bone aging, osteoporotic bone defects

## Abstract

The management of bone defects, particularly in aging populations, remains a major clinical challenge. The immune microenvironment plays an important role in the repair of bone defects and a favorable immune environment can effectively promote the repair of bone defects. However, aging is closely associated with chronic low-grade systemic inflammation, which adversely affects bone healing. Persistent low-grade systemic inflammation critically regulates bone repair through all stages. This review explores the potential of 3D-printed bioceramic scaffolds in bone defect repair, focusing on their capacity to modulate the immune microenvironment and counteract the effects of bone aging. The scaffolds not only provide structural support for bone regeneration but also serve as effective carriers for anti-osteoporosis drugs, offering a novel therapeutic strategy for treating osteoporotic bone defects. By regulating inflammation and improving the immune response, 3D-printed bioceramic scaffolds may significantly enhance bone repair, particularly in the context of age-related bone degeneration. This approach underscores the potential of advanced biomaterials in addressing the dual challenges of bone aging and immune dysregulation, offering promising avenues for the development of effective treatments for bone defects in the elderly. We hope the concepts discussed in this review could offer novel therapeutic strategies for bone defect repair, and suggest promising avenues for the future development and optimization of bioceramic scaffolds.

## 1 Introduction

Bone defects are common tissue injuries that can result from infections, trauma, or tumors (Alonso-Fernández et al., 2023). While bone tissue has some self-healing capacity, critical-sized bone defects, especially in aging people or when the damage surpasses the bone’s natural regenerative ability, are often challenging to heal spontaneously (Tam et al., 2021; Subbiah et al., 2023). The implantation of bone substitute materials is one of the primary methods for treating critical-size bone defects. This includes autografts, allografts, xenografts, and the implantation of tissue engineering scaffolds (Zou et al., 2021). Autografting is regarded as the gold standard for treating critical-size bone defects; however, its limitations include high donor-site morbidity and limited availability (Subbiah et al., 2023). Allografts and xenografts offer alternative options for bone defect repair, but they are limited by immune response complications (Wang Y. et al., 2024). With advancements in 3D printing technology, bone tissue engineering scaffolds enhance surgical precision and safety, while also enabling personalized bone repair. In recent years, 3D-printed scaffolds made from metals, ceramics, and polymers have shown promising preclinical results, with some already being used in clinical settings (Zhao et al., 2020; Zhang J. et al., 2022; Hu et al., 2023). Metal scaffolds are known for their excellent mechanical properties and corrosion resistance, but they lack osseointegration and bioactivity (Lin et al., 2023). In contrast, ceramic scaffolds are more widely used for bone defect repair due to their composition being similar to human bone, providing excellent biocompatibility and good biodegradability (Blázquez-Carmona et al., 2023). Scaffolds made from materials like hydroxyapatite (HA) and tricalcium phosphate (TCP) offer strong osteogenic potential and high biocompatibility. Furthermore, bioactive glass (BG) scaffolds demonstrate high bioactivity and rapid degradation. The properties of these scaffolds can also be improved through various processing techniques, such as phase separation, freeze-drying, solvent casting, gas foaming, electrospinning, and material blending (Gao et al., 2020; Li et al., 2020; Liu et al., 2021; Biscaia et al., 2022; Tommasino et al., 2023; Liu H. et al., 2024).

Bone repair is a complex process that is orchestrated by dynamic interactions between immune cells and bone tissue (Peng et al., 2023; Dai et al., 2024; Liu D. et al., 2024). The immune system plays an essential role in both the physiological and pathological processes of bone tissue, and the modulation of the immune microenvironment is increasingly becoming a favorable target for bone, cartilage and soft tissue regeneration (Xiong et al., 2022; Mi et al., 2024). Bone and the immune system not only share a common microenvironment but also exchange various cytokines and signaling molecules, highlighting the critical role of immune cells in the bone defect repair process (Tsukasaki and Takayanagi, 2019). In postmenopausal women or injury patients, the altered immune status can directly or indirectly result in bone destruction. Polymorphonuclear neutrophils (PMNs) rapidly infiltrate the defect site and release cytokines (e.g., IL-1, TNF-α) to recruit macrophages (Fischer and Haffner-Luntzer, 2022). The differential effects of macrophages on osteoblasts depend on their polarization curves and secreted paracrine factors. It has been shown that various immune cells interact with osteoblasts and osteoclasts either through direct cell-to-cell contact or, more likely, through paracrine mechanisms, in which TNFα increases osteoclast apoptosis and indirectly stimulates osteoclastogenesis through NF-κB ligand receptor activator of kinase (RANKL) produced by B cells (Fischer and Haffner-Luntzer, 2022; Kushioka et al., 2023). Notably, bone aging itself is accompanied by changes in the immune microenvironment and it leads to deteriorating microstructure and function, increasing the risk of osteoporosis and bone defects (Lei et al., 2024; Cui et al., 2024). Aging disrupts bone-immune crosstalk, exacerbating repair challenges and impairing transition to the M2 phenotype. Chronic low-grade inflammation perpetuates M1 polarization and oxidative stress (Mi et al., 2024). Senescent mesenchymal stem cells (MSCs) exhibit diminished proliferative capacity and secrete senescence-associated secretory phenotype (SASP) factors, further inhibiting regeneration (Tong et al., 2024).

3D-printed bioceramic scaffolds can promote bone regeneration by regulating immune microenvironment, cellular senescence and serving as carriers for anti-osteoporosis drugs (Graney et al., 2016). By synergistically inducing bone regeneration and inhibiting bone resorption, these scaffolds offer a promising approach for treating bone defects in osteoporotic patients. This review explores recent advances in the use of 3D-printed bioceramic scaffolds to promote the repair of bone defects by influencing various factors, including their impact on the osteogenic immune microenvironment and the repair of osteoporotic bone defects in the context of bone aging. Diverging from conventional fabrication-focused analyses, we establish a bone immunology-aging nexus, highlighting 3D printing’s unique spatiotemporal precision in coordinating immune-osteogenic repair for aged bone defects. It provides an in-depth analysis of the mechanisms involved and offers directions for future research and improvement of 3D-printed bioceramic scaffolds for bone regeneration following bone defect repair.

## 2 Immune microenvironment during bone defect repair and bone aging

Bone healing progresses through four stages: inflammation, fibrocartilaginous callus formation, bony callus development, and remodeling (Ren et al., 2022). Immune cells primarily influence bone defect repair by affecting the processes of acute and chronic inflammation. Acute inflammation occurs in response to these external stimuli, serving as a crucial signal for the recruitment of immune cells to the injury site. This inflammatory response activates tissue-resident macrophages as well as other local immune cells, initiating an inflammatory cascade that is critical for setting the stage for subsequent tissue repair.

As primary responders to injury sites, polymorphonuclear neutrophils (PMNs) release chemoattractants and cytokines, such as interleukin (IL)-1, IL-6, tumor necrosis factor-α (TNF-α), and macrophage colony-stimulating factor (M-CSF) prior to apoptosis (Granofszky et al., 2018; Gardizani et al., 2019; Zhang et al., 2021). These molecules recruit and activate macrophages, driving chronic inflammatory responses. Macrophages, in particular, play a pivotal role in bone repair and regeneration. They are broadly classified into two functional subtypes: pro-inflammatory M1 macrophages and anti-inflammatory M2 macrophages.

**FIGURE 1:**
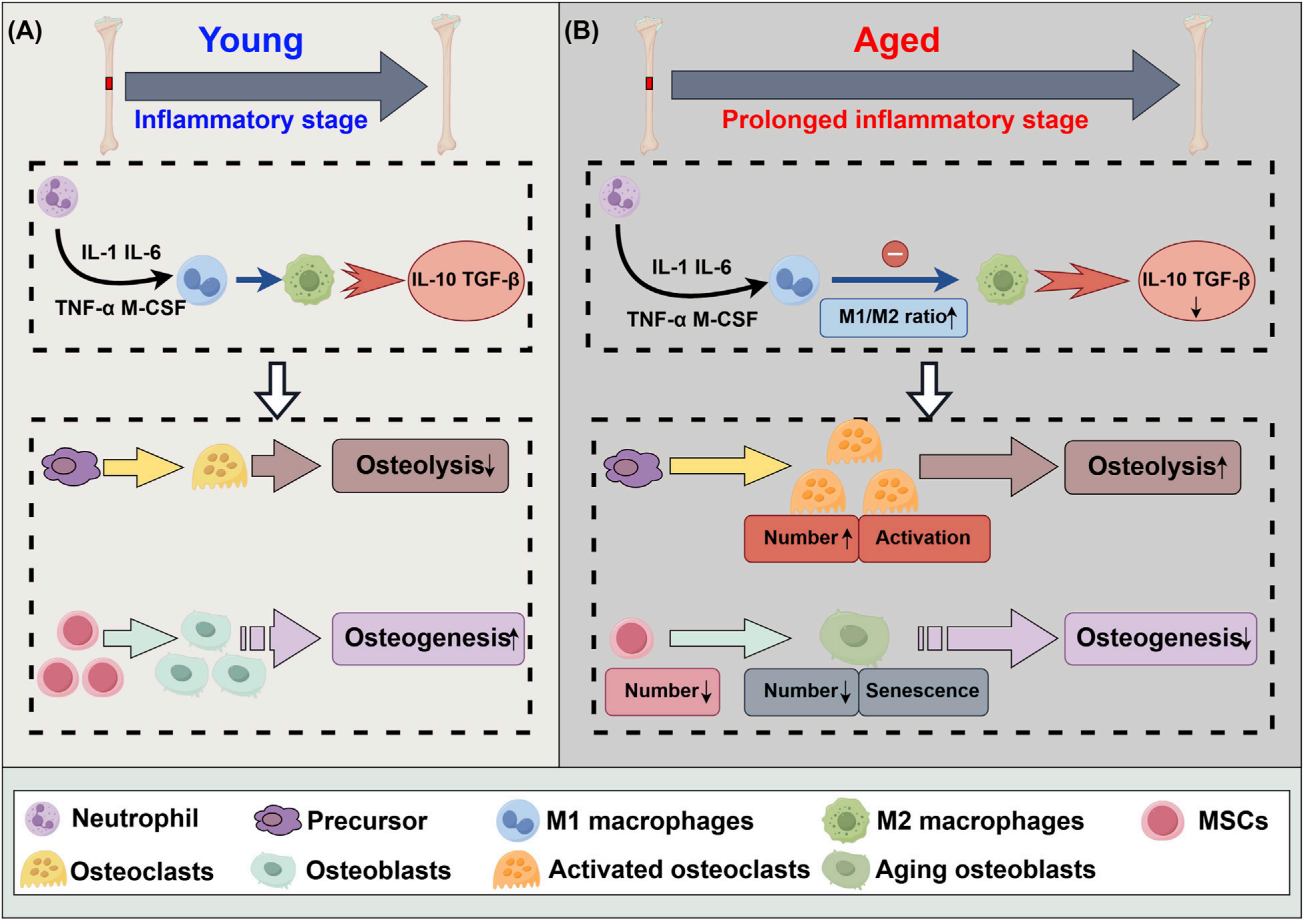
Differences in acute inflammatory phase of bone defect healing between youth and aging. **(A)** The acute inflammatory phase of the bone healing process. **(B)** The acute inflammatory phase of the bone healing process subsequent to senescence. IL-1: interleukin 1, IL-6: interleukin 6, IL-10: interleukin 10, CCL13: chemokine ligand 13, TNF-α: tumor necrosis factor-α, M-CSF: macrophage colony-stimulating factor, TGF-β: transforming growth factor-β, MSCs: mesenchymal stem cells.

M1 macrophages play a major role in the early stages of bone healing. These cells secrete pro-inflammatory factors that promote the breakdown and resorption of damaged bone tissue. While this pro-inflammatory response is essential for initiating repair, prolonged inflammation can impede healing (Holzer-Geissler et al., 2022; Kuan et al., 2025). M2 macrophages, which appear during the later stages of bone repair, are characterized by their anti-inflammatory properties because they secrete IL-10 and transforming growth factor (TGF) (Lv et al., 2017; Lu et al., 2024). With the increase in anti-inflammatory cytokines, mesenchymal stem cells (MSCs) begin to differentiate into osteoblasts, aiding in the regeneration of bone tissue, while osteoclasts work to degrade the damaged bone tissue. Therefore, one of the key factors in enhancing bone regeneration is promoting the transition from M1 to M2 macrophages. This shift not only reduces inflammation but also fosters an environment conducive to bone formation and vascularization, both of which are critical for long-term healing. Figure 1A summarizes the acute inflammatory phase of the bone defect healing process.

The metabolic balance of bone tissue is increasingly disrupted, leading to a decline in bone density and an increased risk of fragility, a phenomenon known as bone aging (Kruger and Wolber, 2016). As individuals age, factors such as telomere shortening, oxidative stress, DNA damage, and epigenetic changes contribute to cellular senescence, reducing the regenerative and repair capabilities of cells (Wang et al., 2022; Kwiatkowska et al., 2023; Harley et al., 2024; Zhu et al., 2024). Senescence is associated with a phenomenon known as “inflammation,” which describes the occurrence of persistent, low-grade systemic inflammation (Li X. et al., 2023). Inflammation resulting from the failure of M1 to M2 polarization increases osteoclast activity, decreases osteoblast formation, and exacerbates bone resorption, ultimately impairing bone healing (Mi et al., 2022; Giraldo-Osorno et al., 2024). Additionally, the number and proliferative capacity of MSCs decline, further aggravating the imbalance between bone formation and resorption, thereby prolonging bone defect healing (Peng et al., 2022). Bone aging serves as a foundation for osteoporosis and other age-related bone diseases. Figure 1B summarizes the acute inflammatory phase of the bone defect healing process subsequent to senescence.

## 3 3D-printed bioceramic scaffolds in immune regulation

**FIGURE 2:**
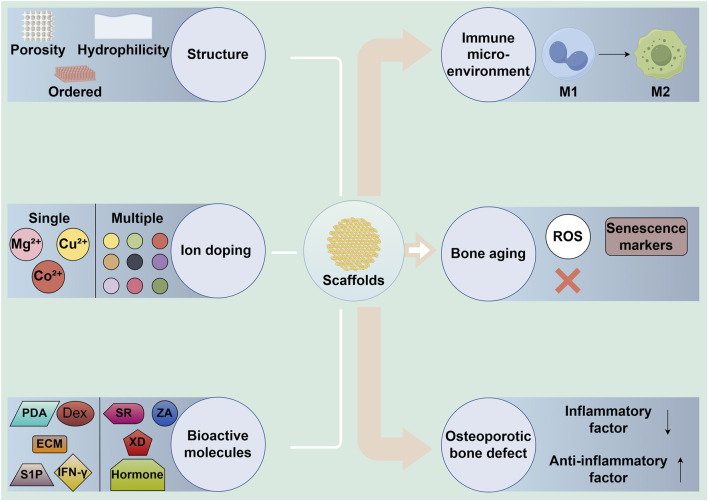
The role of 3D-printed bioceramic scaffolds in regulating immune microenvironment and bone regeneration through structural and bioactive factor modifications. M1: M1 macrophages, M2: M2 macrophages, MSCs: mesenchymal stem cells, ECM: extracellular matrix, IFN-γ: interferon-γ, S1P: sphingosine-1-phosphate, Dex: dexamethasone, PDA: polydopamine.

In the treatment of bone defects, bioceramic scaffolds serve as the structural foundation for tissue regeneration. However, since these scaffolds are recognized by the body as foreign objects, they can trigger an immune rejection response. The nature and intensity of the immune reaction elicited by the scaffold significantly impact the overall success of the treatment. An uncontrolled or excessive immune response could result in chronic inflammation, impaired healing, or even scaffold rejection, while a well-modulated immune response can support tissue regeneration and integration (Wu et al., 2022; Sun et al., 2023). Thus, the scaffold’s ability to regulate the immune microenvironment is paramount to achieving favorable therapeutic outcomes. Changing scaffold structure and bioactive factor modification are crucial approaches for reducing foreign body reactions, modulating the immune microenvironment, and promoting bone regeneration (Figure 2). Table 1 summarizes recent research progress on the use of 3D-printed bioceramic scaffolds to promote bone defect repair by modulating the immune microenvironment.

**TABLE 1:** The application of 3D-printed bioceramic scaffolds in regulating the immune microenvironment for the repair of bone defects.

Year	Team	Scaffold/composition	The roles in immune microenvironment and bone regeneration
2018	Li et al.	PLA/PEG/nHA/Dex	Dex in scaffold suppresses IL-6 and iNOS and promotes M2 macrophage polarization, enhancing bone regeneration.
2018	Li et al.	CaSiO3/β-TCP/IFN-γ	The scaffold releases IFN-γ and Si ions to sequentially polarize macrophages, promoting angiogenesis.
2019	Cao et al.	β-TCP/S1P	The scaffold regulates macrophage response, inhibits inflammation, and promotes osteogenesis.
2019	Lin et al.	Cu/BGC	The Cu2+ ions released from the scaffold promote macrophage polarization, activate the HIF signaling pathway and inhibit the expression of TNF-α and IL-18.
2019	Li et al.	DOPA/BC	The scaffold enhances the paracrine functions of Ad-MSCs, promoting immunomodulation and angiogenesis.
2019	Mansour et al.	DCP/ECM	Bone extracts coating promotes anti-inflammatory M2 macrophage polarization and enhancing osteogenesis.
2020	Ji et al.	PCL/nHA/HPCH/MSCs	The scaffold promotes M2 macrophage polarization, enhances angiogenesis and osteoinduction, and facilitates bone regeneration.
2020	Zhai et al.	LCS	The scaffold promotes macrophage polarization, inhibits TNF-α, IL-6, and IL-1β, and promotes cartilage repair.
2021	Yang et al.	SrFeHA/ PCL	The scaffold promotes the transition of RAW264.7 macrophages and supports the proliferation and differentiation of MC3T3 osteoblasts and HUVECs.
2022	Li et al.	PCL/PEG/HA	The scaffold with 600 μ pore size significantly reduces the FBR and induced more M2 macrophage infiltration, vascular ingrowth, and new bone formation compared to smaller pore sizes.
2022	Pan et al.	Sr2ZnSi2O7	The scaffold promotes macrophage polarization and osteogenesis.
2022	Qi et al.	Mg/TCP	The scaffold promotes the polarization of RAW264.7 and osteogenesis.
2022	Zhang et al.	Ca7Si2P2O16/MSCs/Macrophages	Tai Chi pattern with a 2:1 ratio of MSCs to macrophages is effective in activating anti-inflammatory M2 macrophages and signaling pathways such as BMP-Smad, OSM, and Wnt/β-catenin in MSCs.
2023	Bose et al.	TCP/HA/Garlic extract	The scaffold activates signaling pathways such as BMP-Smad, Oncostatin M, and Wnt/β-catenin in MSCs and promotes osteogenesis.
2023	Deng et al.	SrZn2(PO4)2	The scaffold modulates macrophage polarization, promotes IL-1β, TNF-α, and iNOS, inhibits TGF-β1, IL-1Ra, and CD206, and promotes osteogenesis.
2023	Li et al.	PCL/HA	The scaffold promotes macrophage polarization and enhances osteogenesis through the Wnt/β-catenin pathway.
2023	Xuan et al.	SrZn2(PO4)2	The scaffold promotes the polarization of macrophages and expression of osteogenesis-related markers.
2023	Yu et al.	β-TCP	The scaffold promotes the polarization of macrophages, influences the secretion of BMP-2, TGF-β, and VEGF and promotes osteogenesis.
2024	Xiong et al.	HA	The scaffold promotes the polarization of macrophages, upregulates the production of IFN-β and HIF-1α and enhances bone regeneration.
2024	Xuan et al.	Ca7MgSi4O16	The scaffold pomotes bone regeneration and CD68 + CD206 + M2 macrophage polarization
2024	Geng et al.	Cu/BGC	The scaffold upregulates M2 surface marker Arg-1, inhibits IL-1β and IL-6, promotes IL-4, and enhances osteogenesis.

PLA, poly Lactic Acid; PEG, polyethylene Glycol, nHA, nano hydroxyapatite; Dex, dexamethasone; IL, interleukin; iNOS, inducible nitric oxide synthase; β-TCP, β-tricalcium phosphate; IFN-γ, interferon-γ; S1P, Sphingosine 1-phosphate; TNF-α, Tumor necrosis factor-α; BG, bioactive glass ceramic; DOPA, polydopamine; BC, bioceramic; Ad-MSCs, adipose-derived mesenchymal stem cells; DCP, dicalcium phosphate; ECM, extracellular matrix; PCL, polycaprolactone; HPCH, hydroxypropyl chitin hydrogel; MSCs, mesenchymal stem cells; LCS, lithium calcium silicate, TGF-β, transforming growth factor-β; BMP, bone morphogenetic protein; VEGF, vascular endothelial growth factor.

### 3.1 Impact of scaffold structure on immune regulation

Scaffold structural features directly regulate immune cell interactions. Advancements in 3D printing technology have opened up new possibilities for precisely engineering the structure of bioceramic scaffolds to modulate immune responses.

Ordered scaffolds not only enhance immune cell adhesion and migration but also optimize the immune response by modulating macrophage behavior. Xuan et al. (Xuan et al., 2023) compared ordered Bredigite (BRT-O) scaffolds with random BRT (BRT-R) scaffolds and β-TCP scaffolds and found that BRT-O scaffold could enhance the proliferation, migration, and osteogenic differentiation of bone marrow stromal cells (BMSCs) by promoting M2 macrophage polarization and creating an anti-inflammatory healing environment. This was evidenced by the upregulation of osteogenic markers such as BMP2 and RUNX2, resulting in significantly improved bone regeneration in a rat critical-sized bone defect model. Building on these findings, the authors further investigated the application of the BRT scaffold’s ordered structure in bone graft models (Xuan et al., 2024). The increased expression of anti-inflammatory markers such as CD206 and IL-10 further confirmed that the BRT-O scaffold promotes BMSCs’ osteogenic differentiation and significantly enhances bone regeneration in a rabbit calvarial graft model by inducing M2 macrophage polarization. These two studies highlights the potential of BRT scaffolds in clinical practice and further underscores the critical role of ordered microstructures in immunomodulation. Additionally, Yu et al. (2023) designed a novel gear-inspired 3D-printed bioceramic scaffold with well-ordered surface microstructure. Research indicates that this scaffold can promote M2 macrophage polarization, reduce inflammation, and stimulate the osteogenic differentiation of BMSCs.

Apart from the ordered microstructure, altering the pore size and porosity of the scaffold can also modulate immune responses. The size of pores within the scaffold can significantly impact immune cell behavior, particularly in the modulation of macrophage activity, which is essential for tissue healing and immune regulation. Li et al. (2022) compared polycaprolactone/polyethylene glycol/hydroxyapatite (PCL/PEG/HA) scaffolds with different pore sizes and found that P600 significantly reduced foreign body reactions, induced M2 macrophage infiltration, and promoted vascularization and bone regeneration. Also, macrophage polarization may be related to the MyD88 protein. In another study, Xiong et al. (2024) fabricated three different pore-sized hydroxyapatite (HA) bioceramic scaffolds to investigate the systematic effects of pore size on the immune microenvironment. The results indicate that the 600 μm pore-sized scaffold most effectively promoted macrophage M2 polarization and improved the inflammatory response by upregulating interferon-β and HIF-1α production.

Another important strategy for regulating the immune response is to alter the hydrophilicity of the scaffold surface. Compared to hydrophilicity, the hydrophobicity of the scaffold surface reduces cell adhesion and bioactivity, significantly impacting the osteogenic capacity of bioceramic scaffolds (Meng et al., 2022). Li et al. (2023a) enhanced the hydrophilicity of PCL/HA scaffolds through alkali treatment and found that the scaffold significantly reduced the foreign body response and promoted M2 macrophage polarization. In addition, it was found that the regulation of osteogenesis by hydrophilic surface scaffolds may be related to the Wnt/β-catenin signaling pathway.

The above research results show that modifying the structure of 3D-printed bioceramic scaffolds can guide macrophage polarization toward an anti-inflammatory phenotype and enhance the activity of key cell types such as BMSCs, thereby actively facilitating tissue repair and regeneration. However, the balance between the bioactivity and mechanical properties of the scaffold will need to be further optimized in the future.

### 3.2 Impact of scaffold bioactive factor modification on immune regulation

In addition to the structural characteristics of 3D-printed bioceramic scaffolds, scaffold bioactive factor modification also plays a significant role in modulating the immune response during bone regeneration.

#### 3.2.1 Ion doping

Bioactive bioceramics interact with the cellular environment via surface contact and ion release into the tissue microenvironment. The various ions released from the scaffold, such as magnesium (Mg^2+^), zinc (Zn^2+^), and copper (Cu^2+^), have been shown to modulate the immune microenvironment, particularly by influencing macrophage polarization and promoting bone regeneration (Yang J. et al., 2023; Li et al., 2024; Sun et al., 2024).

Mg^2+^ is a key component in bone tissue, contributing to bone metabolism and maintaining homeostasis (Qiao et al., 2021). A deficiency in Mg^2+^ can lead to insufficient bone formation and metabolic disturbances in bone. Therefore, the incorporation of Mg^2+^ into bioceramic scaffolds to promote osteogenesis and regulate the immune microenvironment has garnered widespread attention. For instance, Qi et al. (2022) developed a 3D-printed magnesium-doped β-TCP scaffold for bone regeneration. Varying MgO contents (0%, 1%, 3%, 5%) were tested, with 3% Mg-TCP showing the best biological performance. *In vitro* experiments have demonstrated that 3 Mg-TCP enhanced the osteogenic and angiogenic differentiation of BMSCs and endothelial progenitor cells. It also promoted M2 macrophage polarization, supporting tissue regeneration. *In vivo* experiments have demonstrated that 3 Mg-TCP demonstrated superior bone repair capabilities in a rabbit femoral defect model. Cu^2+^ is another important ion for regulating immune responses and promoting tissue regeneration. Lin et al. (2019) demonstrated that copper-incorporated bioactive glass ceramics (Cu-BGC) enhance cartilage and bone regeneration by promoting chondrocyte proliferation and inducing macrophage polarization towards the anti-inflammatory M2 phenotype. The potential mechanism may involve released Cu^2+^ stimulating cartilage immune responses through the HIF-1α pathway and inhibiting tissue inflammation. In a subsequent study, Geng et al. (2024) found that Cu-BGC significantly inhibited the expression of pro-inflammatory cytokines (e.g., IL-1β and IL-6) by inducing M2 macrophage polarization, while upregulating anti-inflammatory cytokines (e.g., IL-4), indicating its anti-inflammatory effects. Additionally, Cu-BGC significantly reduced the expression of matrix-degrading enzymes (e.g., MMP3, MMP13), suggesting that Cu-BGC effectively protects cartilage matrix from degradation in inflammatory conditions. The above research indicates that bioceramic scaffolds with single-ion doping can promote bone regeneration by enhancing macrophage polarization, which holds significant value in the treatment of bone defects.

Based on the foundation of single ion doping, the complementary doping of multiple beneficial elements to enhance biological effects is now considered a promising new strategy. Yang et al. (2021) investigated the synergistic biological effects of Sr^2+^/Fe^3+^ by employing extrusion-based low-temperature 3D printing technology to fabricate SrFeHA/PCL scaffolds, comparing them with scaffolds doped solely with Sr10HA and Fe10HA. The results indicated that the synergistic effect of Sr^2+^/Fe^3+^ not only modulates the immune response by promoting M2 macrophage polarization but also enhances the functionality of MC3T3 osteoblasts and HUVECs. Zhai et al. (2020) investigated whether lithium calcium silicate (LCS) bioceramic scaffolds could promote the repair and regeneration of cartilage tissue by inducing the differentiation of macrophages in a specific direction. The experimental results showed that LCS scaffolds promote M2 macrophage polarization by reducing the activity of inflammatory-related genes TNFα, IL-6, and IL-1β, while enhancing the expression of the IL-10 gene, thereby promoting the growth of chondrocytes. Pan et al. (2022) developed 3D-printed Sr_2_ZnSi_2_O_7_ (SZS) scaffolds. Research found that these scaffolds possess good immunomodulatory functions, modulating the inflammatory response of macrophages by releasing bioactive ions. This is specifically manifested by promoting an immune environment conducive to healing and reducing the release of inflammatory factors. However, the issues of rapid degradation rate and low mechanical strength remain challenges to be addressed in the future.

The multi-ion doping method still faces certain challenges in precisely controlling the proportions and distribution of each dopant ion. In contrast, the single-phased bioceramics with a fixed composition offer greater stability. Deng et al. (2023) compared 3D-printed strontium-zinc-phosphate (SZP) bioceramic scaffolds with β-TCP scaffolds and found that SZP scaffolds possess superior osteogenic, angiogenic, and antibacterial properties, and promote M2 macrophage polarization.

The above research results reveal the significant potential of ion-doped bioceramic scaffolds in immune modulation. By releasing specific bioactive ions, these scaffolds can effectively modulate the inflammatory microenvironment of macrophages, promoting the phenotypic transformation of macrophages with anti-inflammatory properties. This transformation is crucial for facilitating the healing process of bone defects. However, the synergistic effects between ions and the underlying mechanisms of their interactions still need to be further explored, and the accuracy and stability of the proportion and distribution of doped ions require enhancement.

#### 3.2.2 Loading of bioactive molecules

Bioceramic scaffolds loaded with bioactive molecules not only provide structural support for tissue regeneration but also promote tissue repair by leveraging various immunomodulatory mechanisms.

The extracellular matrix (ECM), composed of organic components like proteins, polysaccharides, and growth factors, plays a pivotal role in regulating cellular behavior and directing tissue regeneration and development (Wang J. et al., 2024). Mansour et al. (2019) demonstrated that coating dicalcium phosphate bioceramic scaffolds with bone ECM extracts, especially calcium-binding E-extract, enhances bone regeneration through immunomodulation. E-extract-coated scaffolds reduced inflammatory responses, promoted anti-inflammatory macrophage activity, and significantly improved bone formation in rat tibial defects. The study also shows that 3D-printed hydrogel scaffolds incorporating E-extract support better bone regeneration, suggesting future applications for personalized, ECM-based scaffolds in complex bone repair. However, whether this process is related to the conversion of M1 to M2 macrophages remains to be verified.

Interferon-γ (IFN-γ) and sphingosine-1-phosphate (S1P), among others, regulate host immune responses through different mechanisms to promote bone regeneration. Li et al. (2018b) presented a 3D-printed calcium silicate-β-tricalcium phosphate (CaSiO_3_-β-TCP) scaffold loaded with IFN-γ. The research results indicate that the scaffold sequentially activates M1 and M2 macrophage polarization: M1 for early inflammation and M2 for tissue repair. *In vitro* experiments have demonstrated that it enhances macrophage-driven angiogenesis and bone regeneration by increasing the secretion of VEGF and PDGF-BB. *In vivo* experiments have demonstrated that the scaffold improves blood vessel formation and bone healing in a mouse model. Additionally, S1P is an important immunomodulatory molecule that, when coated on β-tricalcium phosphate scaffolds, effectively inhibits inflammation and promotes osteogenesis. S1P plays a crucial role in bone formation by regulating macrophage migration and the expression of inflammation-related genes. Cao et al. (2019) evaluated 3D-printed β-TCP scaffolds coated with S1P for immunomodulation and bone regeneration. The research results indicate that the S1P-coated scaffolds reduce inflammation by downregulating pro-inflammatory cytokines and promote osteogenesis by upregulating osteogenic genes like OCN, OPN, and RUNX2. Additionally, this scaffold also promotes the differentiation of BMSCs into osteoblasts. This dual action of reducing inflammation and enhancing bone regeneration makes S1P-coated scaffolds a promising option for treating large bone defects.

Dexamethasone (Dex), as a potent glucocorticoid, not only promotes osteogenesis but also effectively controls local inflammation due to its immunosuppressive properties. Li et al. (2018a) developed a composite scaffold made from PLA, PEG, nHA, and Dex using 3D printing for bone regeneration and found that Dex release from the scaffold modulated inflammation by promoting M2 macrophage polarization and enhanced osteogenesis by increasing late alkaline phosphatase secretion and calcium deposition. In a rat calvarial defect model, the scaffold improved bone regeneration without adverse effects on vital organs.

Due to its excellent biocompatibility and degradability, polydopamine (PDA) shows great potential for applications in the field of tissue engineering. Li et al. (2019) conducted a comparative study by culturing adipose-derived mesenchymal stem cells (Ad-MSCs) on BC scaffolds and PDA-modified BC scaffolds and found that polydopamine biomimetic coating significantly enhanced the paracrine capabilities of mesenchymal stem cells. Specifically, Ad-MSCs cultured on polydopamine-modified BC scaffolds (DOPA-BC) were able to secrete a greater amount of immunomodulatory factors. This phenomenon promoted the polarization of M2-type macrophages, thereby playing a positive role in immunomodulation.

3D-printed bioceramic scaffolds loaded with immune cells have shown unique immunomodulatory potential in bone tissue engineering. Using 3D printing technology to load macrophages or MSCs into bioceramic scaffolds can create a complex network of cellular interactions. The spatial distribution and arrangement of these cells on the scaffold can significantly affect their immunomodulatory functions. For example, Zhang et al. (2022a) in order to observe the intercellular “cross-talk” between macrophages and MSCs within a three-dimensional structure, used digital light processing-based 3D printing technology to create a multi-channel honeycomb-like bioceramic scaffold. The study results showed that the “Taiji” pattern with a 2:1 ratio of MSCs to macrophages more effectively stimulated M2 polarization and promoted the osteogenic differentiation of MSCs. They also found that this effect may be associated with the activation of the BMP-Smad, Oncostatin M, and Wnt/β-catenin signaling pathways.

Bioceramic scaffolds loaded with bioactive molecules represent a cutting-edge approach in bone regeneration and immunomodulation, effectively reducing inflammatory responses and regulating macrophage polarization. Additionally, the rational adjustment of the spatial arrangement and distribution of immune cells can enhance immunomodulatory capabilities. However, research on the loading of multiple bioactive molecules, as well as their spatial distribution and arrangement, is insufficient, and further studies are needed in the future.

## 4 3D-printed bioceramic scaffolds in bone aging and osteoporosis

**FIGURE 3:**
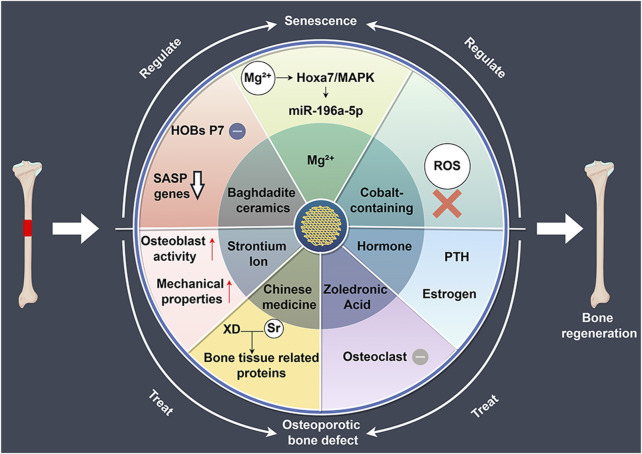
The role of 3D-printed bioceramic scaffolds in regulating bone regeneration in aging and osteoporosis environments. HOBs: human primary osteoblast-like cells, SASP: senescence-associated secretory phenotype, XD: Xu Duan, ROS: reactive oxygen species, PTH: parathyroid hormone.

With the occurrence of bone aging, the incidence of age-related diseases such as osteoporosis has been rising year by year, posing a significant challenge to public health. Osteoporosis is a systemic degenerative bone disease associated with aging, characterized by reduced bone mass and deterioration of bone microstructure, thereby leading to increased bone fragility and risk of fracture, known as osteoporotic fractures (Patsch et al., 2011). 3D-printed bioceramic scaffolds can be used to treat bone defects in an aging environment by modulating bone aging and delivering drug therapies for osteoporotic bone defects (Figure 3). Table 2 summarizes recent research advancements in the modulation of bone aging and the treatment of osteoporotic bone defects using 3D-printed bioceramic scaffolds.

**TABLE 2:** The application of 3D-printed bioceramic scaffolds in treating aged and osteoporotic bone defects.

Year	Team	Scaffold composition	The roles in bone aging and treating osteoporosis
2015	Tripathi et al.	CDHA/Quercetin	Quercetin, stably released from CDHA scaffolds, promotes the proliferation of pre-osteoblast cells (MC3T3-E1) and inhibits the proliferation of osteoclasts to treat osteoporosis.
2016	Meininger et al.	Sr-Mg3(PO4)2	Strontium ions promote the proliferation of osteoblasts and inhibit the proliferation of osteoclasts to treat osteoporosis.
2019	Gómez-Cerezo et al.	MBG/PCL/ZA	The released ZA can inhibit the proliferation and differentiation of osteoclasts to treat osteoporosis.
2020	Lu et al.	Baghdadite	Baghdadite ceramics can provide an anti-senescent microenvironment that prevents the induction of cellular senescence in late passaged HOBs and modulates the secretory profiles of these cells, reducing their pro-senescent paracrine effects.
2021	Kao et al.	SrCS/XD	The addition of Xu Duan to the scaffolds enhance cell proliferation and the secretion of osteogenic-related proteins to treat osteoporosis.
2022	Deng et al.	AKT/HNPs/HMPs	The scaffold scavenges ROS, promotes the proliferation of chondrocytes, and facilitates the osteogenic differentiation of BMSCs.
2023	Ran et al.	Mg-Nd-Zn-Zr/ZA	The osteogenic effect of Mg and the osteoclast inhibition effect of ZA facilitate the repair of osteoporotic bone defects.
2023	Ren et al.	PMBG/TCP/PTH	PMBG/TCP scaffolds, coordinated with PTH (1-34), can bidirectionally regulate bone homeostasis, promoting bone formation while inhibiting bone resorption.
2023	Shu et al.	Zn/Co-MOF/β-TCP	The scaffold scavenges ROS and promotes cartilage regeneration.
2024	Codrea et al.	HA/PCL/SrHA	The composite scaffold possesses excellent osteogenic potential and cell compatibility, and is capable of releasing strontium ions for the treatment of osteoporosis.
2024	Qi et al.	Ca2MgSi2O7	The scaffold promotes osteogenesis and delays the senescence of O-BMSCs through the exosome-mir-196a-5p/Hoxa7/MAPK signaling axis.
2024	Shu et al.	Co-ClAP	The scaffold scavenges ROS and promotes cartilage regeneration.

CDHA, calcium deficient hydroxyapatite; MBG, mesoporous bioactive glass; PCL, e-Polycaprolactone; ZA, zoledronic acid, Baghdadite; Ca3ZrSi2O9, HOBs, human osteoblast-like cells; XD, XuDuan, AKT, Akermanite; HNPs, hair-derived antioxidative nanoparticles; HMPs, hair-derived antioxidative microparticles; ROS, reactive oxygen species; BMSCs, bone marrow mesenchymal stem cells; PMBG, photo-cured mesoporous bioactive glass; TCP, tricalcium phosphate; PTH, parathyroid hormone; MOF, bimetallic organic framework; O-BMSCs, osteoporotic bone mesenchymal stem cells; Co-ClAP, Cobalt-Incorporated Chloroapatite.

### 4.1 Bioceramic scaffolds regulate bone aging

3D-printed bioceramic scaffolds with tailored structures and compositions effectively support bone regeneration in aging microenvironments. Lu et al. (2020) compared Baghdadite scaffolds with HA/TCP scaffolds and found that Baghdadite scaffolds significantly reduced the expression of senescence markers (P16, P21) in P7 human primary osteoblast-like cells (HOBs), as well as senescence-associated secretory phenotype markers, such as IL-1α, TNF-α, and IL-6. Additionally, Baghdadite scaffolds decreased the paracrine effects of P7 HOB secretions in inducing senescence in young cells. Notably, Baghdadite scaffolds also improved mitochondrial function. These findings suggest that Baghdadite ceramics are a promising biomaterial capable of creating an anti-senescence and pro-regenerative microenvironment.

In addition, Mg^2+^ can not only regulate the immune microenvironment but also play a significant role in modulating the aging microenvironment. Qi et al. (2024) in order to investigate the effects of magnesium ions on osteoporosis and osteogenesis, fabricated Akermanite bioceramics (Akt) scaffolds enriched with Mg ions. The research found that magnesium-rich bioceramics can effectively improve bone regeneration impairments caused by aging by regulating the osteogenic differentiation and angiogenesis of BMSCs. Mg^2+^ enhances bone regeneration by targeting the Hoxa7/MAPK signaling axis, which modulates the secretion of miR-196a-5p in exosomes, thereby influencing the expression of osteogenesis-related genes in the aging microenvironment. This provides a potential strategy for bone regeneration in an aging microenvironment.

Oxidative stress plays a critical role in aging and bone tissue degeneration, particularly in the process of bone tissue regeneration. Reactive oxygen species (ROS)-induced damage to cellular components is considered one of the key mechanisms promoting aging (He et al., 2024). Similar to other tissues, the accumulation of oxidative stress-induced cellular and tissue damage in bone increases with age, leading to a decline in bone cell function and senescence (Xu et al., 2020). Mitochondria are the primary source of ROS. With advancing age, mitochondrial ATP production capacity declines, and the efficiency of antioxidant defense systems also diminishes, resulting in elevated intracellular ROS levels (Cheng and Ristow, 2013). This oxidative stress not only damages macromolecules such as cell membranes, DNA, and proteins but also triggers a range of age-related pathological changes, including chronic inflammation, neurodegenerative diseases, cardiovascular diseases, and diabetes (Kello et al., 2020; Fang et al., 2022; Hanchang et al., 2022; Yang Y. M. et al., 2023; Prata et al., 2024). Using 3D-printed bioceramic scaffolds to alleviate oxidative stress may offer new strategies for slowing aging and preventing related diseases. Shu et al. (2023) developed Zn/Co-MOF-functionalized β-TCP scaffolds to enhance osteochondral regeneration in osteoarthritis. The research results indicate that the scaffold has a broad ability to clear ROS and promotes osteogenic and chondrogenic differentiation. In subsequent research, Shu et al. (2024) developed Co-ClAP/PLGA composite scaffolds using 3D printing technology. The research indicates that this scaffold possesses antioxidant characteristics, capable of neutralizing excessive ROS in inflammatory environments, thereby maintaining cell proliferation, adhesion, and differentiation, and concurrently promoting the regeneration of cartilage and subchondral bone. The above results demonstrate that these two cobalt-containing bioceramic scaffolds not only exhibit strong antioxidant properties, significantly clearing ROS, but also promote osteogenesis. Future research should focus on enhancing the antioxidant properties of the scaffold while improving osteogenic capacity.

3D-printed bioceramic scaffolds play a crucial role in combating bone aging. They significantly promote bone tissue regeneration under aging conditions by reducing the expression of aging markers and modulating the activity of osteogenesis-related genes in a aging environment. Additionally, these scaffolds exhibit excellent antioxidant properties, effectively neutralizing excessive ROS in inflammatory environments, thereby combating the aging process triggered by oxidative stress.

### 4.2 Drug-loaded bioceramic scaffolds treat osteoporotic bone defects

The primary factors contributing to osteoporosis include intrinsic factors related to natural aging, which heighten bone resorption and reduce bone formation, as well as external factors, such as long-term glucocorticoid use, which further disrupt bone microarchitecture and lead to osteoporosis. Over the past few decades, various pharmacological treatments have been developed to treat osteoporotic bone defects, broadly categorized into anti-resorptive agents (which prevent bone breakdown) and anabolic agents (which stimulate bone formation) (Fixen and Fixen, 2022; Shane et al., 2022; Hartz et al., 2024). In recent years, advances in 3D printing technology have revolutionized the field of bone regeneration by enabling the development of bioceramic scaffolds as drug carriers.

#### 4.2.1 Strontium ion

Strontium ions, the active component of strontium ranelate, enhance bone formation while suppressing resorption. Codrea et al. (2024) utilized 3D printing technology to fabricate scaffolds made of PCL and strontium-substituted hydroxyapatite (SrHA) with the aim of enhancing the recovery of osteoporotic bone defects. The results indicated that scaffolds incorporating SrHA exhibited improved mechanical properties and osteogenic potential, suggesting their potential application in the treatment of osteoporotic bone defects. Meininger et al. (2016) fabricated 3D-printed strontium-substituted magnesium phosphate (SrMPC) scaffolds designed for bone regeneration. These biodegradable scaffolds show good mechanical strength (36.7 MPa compressive) and a porous architecture ideal for bone ingrowth. *In vitro* studies confirmed controlled degradation and a sustained release of Sr2+, which promotes osteoblast activity. In both studies, it has been demonstrated that strontium-doped bioceramic scaffolds possess superior mechanical properties, offering significant advantages in the repair of osteoporotic bone defects. However, the stability of strontium release rates still needs to be further improved.

#### 4.2.2 Zoledronic acid

Zoledronic acid (ZA), a third-generation bisphosphonate with high affinity for bone tissue, is a first-line treatment for osteoporotic bone defects. It inhibits osteoclast differentiation and induces osteoclast apoptosis, thereby suppressing bone resorption. Ran et al. (2023) developed a 3D-printed biodegradable Mg^2+^ scaffold with a ceramic coating loaded with ZA for the treatment of osteoporotic bone defects. Studies have shown that the ZA coating not only reduces the corrosion rate of the scaffold but also achieves precise and slow drug release. Furthermore, due to the combined action of Mg^2+^ and ZA, the scaffold promotes bone formation while inhibiting osteoclast activity, effectively facilitating the repair of osteoporotic bone defects. In another study, Gómez-Cerezo et al. (2019) developed mesoporous bioactive glass (MBG)/PCL scaffolds for bone regeneration in osteoporotic sheep. The scaffold demonstrated good biocompatibility *in vivo*, promoting both bone formation and angiogenesis. Nonetheless, when loaded with 1% ZA, the scaffold induced a strong inflammatory response and impaired bone healing. The above results indicate that bioceramic scaffolds loaded with ZA show great potential in promoting the repair of osteoporotic bone defects, but further research and optimization are needed in terms of drug dosage to achieve better therapeutic effects.

#### 4.2.3 Chinese medicine

Traditional Chinese medicine has long been valued for its holistic approach to health, and its integration with modern medical technologies presents an innovative avenue for treating osteoporotic bone defects. Kao et al. (2021) investigated the efficacy of 3D-printed scaffolds made from poly-ε-caprolactone, strontium-doped calcium silicate, and Xu Duan (a traditional Chinese medicine) for bone regeneration in osteoporosis. The results indicate that XD and Sr ions have a synergistic effect. *In vitro* and *in vivo*, the scaffold, especially those with a higher concentration of strontium (XD10), can increase the secretion of bone tissue related proteins and promote the repair of osteoporotic bone defects. These scaffolds promote bone healing by enhancing osteoblast activity and bone mineralization. This approach opens up new possibilities for combining traditional medicine with advanced materials, offering a promising strategy for treating osteoporotic bone defects. However, research on the combination of traditional Chinese medicine with 3D-printed bioceramic scaffolds is insufficient, and future studies should place greater emphasis on the use of traditional Chinese medicine in bone tissue engineering and the treatment of osteoporotic bone defects.

#### 4.2.4 Hormone

Anabolic bone therapies aim to enhance bone mass by stimulating bone formation. One widely used strategy is the intermittent activation of parathyroid hormone (PTH), which encourages osteogenesis by promoting osteoblast activity. Ren et al. (2023) studied a 3D-printed scaffold composed of photocurable mesoporous bioactive glass (PMBG) and TCP loaded with PTH (1-34). The research findings indicate that the scaffold, in conjunction with PTH (1-34), regulates bone homeostasis bidirectionally by activating the Wnt/β-catenin pathway and inhibiting fibroblast activation protein. This study underscores the potential of combining advanced materials like PMBG and TCP with anabolic agents such as PTH to create scaffolds that not only support bone regeneration but also modulate the underlying cellular mechanisms to optimize healing. Further research is necessary to fully elucidate the underlying molecular pathways and optimize the therapeutic potential of PTH in clinical applications for the treatment of osteoporotic bone defects.

Postmenopausal estrogen deficiency accelerates bone resorption, driving significant bone loss. Hormone replacement therapy has been a key method for preventing and treating osteoporotic bone defects in women (Chen et al., 2020). Tripathi et al. (2015) developed 3D-printed calcium-deficient hydroxyapatite scaffolds loaded with quercetin for the treatment of osteoporotic bone defects. The research findings indicate that the quercetin-loaded scaffolds significantly enhanced pre-osteoblast cell (MC3T3-E1) activity and suppressed osteoclast proliferation, outperforming traditional treatments like alendronate. The potential advantage of this type of scaffold may be its capacity to offer more effective solutions for the treatment of osteoporotic bone defects, particularly in the treatment of postmenopausal osteoporotic bone defects.

## 5 Discussion

As the population ages, bone defects from various causes continue to be a clinical challenge. Due to their excellent biocompatibility and potential for personalized design, 3D-printed bioceramic scaffolds show great promise in addressing these defects. In this review, we aim to explore the critical role of 3D-printed bioceramic scaffolds in the repair of bone defects, highlighting their ability to regulate the immune microenvironment, combat bone aging, and their application in the regeneration of osteoporotic bone defects. By optimizing scaffold design and modifying the scaffold, it is possible to effectively regulate the polarization state of macrophages, reduce inflammatory responses, and promote the process of bone regeneration (Mansour et al., 2019; Xuan et al., 2023). Also, 3D-printed bioceramic scaffolds exhibit good antioxidant properties, capable of neutralizing excessive ROS in the inflammatory environment, and combating aging phenomena caused by oxidative stress (Shu et al., 2024). More importantly, 3D-printed bioceramic scaffolds not only reduce the expression of senescence markers but also promote bone regeneration in a senescent environment (Lu et al., 2020). By delivering osteoporosis drugs, bioceramic scaffolds have also shown a promising application prospect in the treatment of osteoporotic bone defects (Gómez-Cerezo et al., 2019; Kao et al., 2021).

In comparison with conventional artificial molding or phase separation techniques, 3D printing technology facilitates the precise fabrication of complex structures for personalised defect repair and immunomodulation, as well as the integration of multifunctional active ingredients during the fabrication process to further promote bone defect repair by modulating the immune microenvironment and bone ageing. Although 3D-printed bioceramic scaffolds have made significant breakthroughs in the treatment of bone defects, there are still several key issues that need in-depth exploration. Firstly, the inadequate alignment of materials’ biocompatibility, degradability, and mechanical properties with bone defects hinders clinical translation. Secondly, current research lacks a detailed and in-depth analysis of the mechanisms by which 3D-printed bioceramic scaffolds induce macrophage polarization. Research on the role of 3D-printed bioceramic scaffolds in regulating cellular senescence remains limited. It is recommended that subsequent studies investigate the manner in which the surface topology of 3D-printed bioceramic scaffolds and the microenvironment loaded with actives coordinates macrophage polarisation with cellular senescence. Such studies should also explore the synergistic intervention of conventional RANKL, NF-κB, TGF-β/Smad, p53, and other pathways, as well as explore new possible mechanisms.

The process of bone repair is a complex physiological process that involves not only the repair of the bone itself but also has a close relationship with surrounding tissues such as muscles and blood vessels (Zhai et al., 2021; Toita et al., 2024). The importance of blood vessels in bone regeneration lies in their creation of a microenvironment conducive to bone regeneration, which provides sufficient nutrients, growth factors, and oxygen for bone tissue repair. Vascular endothelial growth factor (VEGF) is a key regulatory factor in angiogenesis, promoting the migration and proliferation of endothelial cells through the regulation of osteogenic growth factor release and paracrine signaling, thereby indirectly promoting the osteogenic process (Burger et al., 2022). Studies have found that VEGF-decorated crystalline SiHA scaffolds can effectively treat osteoporotic bone defects (Casarrubios et al., 2020). Enhanced vascularization observed in VEGF-decorated scaffolds not only aids in bone regeneration but also in the overall integration of the scaffold with surrounding tissues. Therefore, an ideal 3D-printed bioceramic scaffold should induce vascularization to optimize the bone repair process. Additionally, studies have shown that skeletal muscle also plays a crucial role in the process of bone repair. The expression of bone morphogenetic protein 2 (BMP-2) in autologous muscle tissue significantly enhances its ability in bone regeneration, which has a significant therapeutic effect on the treatment of bone defects (Kong et al., 2020).

Therefore, future scaffold designs should take a comprehensive approach, considering the regulation of the immune microenvironment, the mitigation of bone aging, the promotion of vascularization, and the modulation of surrounding muscle tissues. Regulating M1/M2 polarization will be one of the key factors in promoting bone defect repair. M1 macrophages are usually associated with inflammatory responses, and inhibiting M1 polarization or inducing M2 macrophage polarization helps reduce chronic inflammation and promote tissue repair. M2 macrophages not only play a role in anti-inflammatory processes but also promote bone reconstruction and vascularization by secreting growth factors and cytokines. By optimizing the surface properties and microstructure of the scaffold, designing materials that promote M2 macrophage polarization will help improve immune responses and accelerate bone repair processes. Additionally, bone aging is a critical factor influencing the bone defect repair process. The design of scaffold materials should have characteristics that combate or even reverse bone aging, such as enhancing bone density and rejuvenating senescent bone cells. Vascularization is another important factor for successful scaffold implantation, as a robust vascular network is essential for supplying the necessary nutrients and oxygen to the repair site, thereby facilitating bone tissue regeneration. In addition, the control of surrounding muscle tissues is also an indispensable dimension in the bone defect repair process, as muscle function recovery has a direct impact on the bone repair. Therefore, the design of future bioceramic scaffolds should integrate these multifaceted biological needs, promoting the research and clinical application of multifunctional scaffolds.

## 6 Conclusion

3D-printed bioceramic scaffolds demonstrate significant potential in regulating the immune microenvironment and mitigating bone aging. By optimising scaffold structures and incorporating bioactive factors, these scaffolds can modulate the immune microenvironment, effectively target specific immune pathways, and promote tissue healing and osteogenic differentiation. In addition, the modified bioceramic scaffolds reduce cellular senescence and oxidative stress, enhance bone regeneration and serve as carriers for anti-osteoporotic drugs, further contributing to bone regeneration. In summary, bioceramic scaffolds present innovative solutions for the treatment of bone defects and promise to provide multiple strategies for maintaining bone health.
